# Association of *TLR4* and *TLR9* polymorphisms and haplotypes with cervical cancer susceptibility

**DOI:** 10.1038/s41598-019-46077-z

**Published:** 2019-07-05

**Authors:** Nilesh O. Pandey, Alex V. Chauhan, Nitin S. Raithatha, Purvi K. Patel, Ronak Khandelwal, Ajesh N. Desai, Yesha Choxi, Rutul S. Kapadia, Neeraj D. Jain

**Affiliations:** 1 P D Patel Institute of Applied Sciences, Charotar University of Science and Technology (CHARUSAT), Changa, Anand, India; 2 Department of Obstetrics and Gynaecology, Pramukh Swami Medical College, Shree Krishna Hospital, Karamsad, Anand, India; 3 Department of Obstetrics and Gynaecology, Sir Sayajirao General Hospital and Medical College, Vadodara, India; 4 Department of Obstetrics & Gynaecology, GMERS Medical College and Hospital, Ahmedabad, India

**Keywords:** Cervical cancer, Cervical cancer, Cancer genetics

## Abstract

Single nucleotide polymorphisms (SNPs) in *TLR* genes may serve as a crucial marker for early susceptibility of various cancers including cervical cancer. The present study was therefore designed to ascertain the role of *TLR4* and *TLR9* SNPs and haplotypes to hrHPV infection and cervical cancer susceptibility. The study included 110 cervical cancer biopsies and 141 cervical smears from age-matched healthy controls of Gujarati ethnicity of Western India. hrHPV 16 and 18 were detected using Real-time PCR. Eight SNPs, four each in *TLR4* and *TLR9* were analyzed using Polymerase Chain Reaction-Restriction Fragment Length Polymorphism and Allele-Specific PCR. HPV 16 and 18 were detected in 68% cervical cancer cases. *TLR4* rs4986790, rs1927911 and *TLR9* rs187084 showed association with HPV 16/18 infection. CC and CT genotypes of *TLR4* rs11536889 and rs1927911 respectively, and TC, CC genotypes of *TLR9* rs187084, as well as minor alleles of *TLR4* rs4986790 and *TLR9* rs187084, were associated with the increased risk of cervical cancer. Stage-wise analysis revealed *TLR9* rs187084 and rs352140 to be associated with early-stage cancer. *TLR4* haplotype GTAC and *TLR*9 haplotype GATC were associated with the increased risk of cervical cancer while *TLR4* haplotype GCAG was associated with the decreased risk. *TLR4* haplotype GCAG and *TLR9* haplotype GATC showed association with increased susceptibility to hrHPV infection. In conclusion, the present study revealed association of *TLR4* and *TLR9* polymorphisms and haplotypes with hrHPV infection and cervical cancer risk. Further evaluation of a larger sample size covering diverse ethnic populations globally is warranted.

## Introduction

With respect to gender-specific cancers, cervical cancer is the next major cause of global cancer deaths among women, after the cancer of the breast, wherein India accounts for almost one-fourth of total cervical cancer-related mortalities^1^. Human papillomavirus (HPV) infection is considered as the most vital event in the development and progression of cervical cancer, as the HPV DNA has been detected in almost all of the cervical tumors globally^2^. With more than 200 HPV types known till date, fifteen (16, 18, 31, 33, 35, 39, 45, 51, 52, 56, 58, 59, 68, 73, and 82) designated as high-risk types have been found to be associated with cervical cancer and precancerous lesions^3^. Of the various high-risk types, the combined prevalence of HPV 16 and 18 is estimated to be approximately 70% worldwide^4^. The key targets of HPV are epithelial cells of the skin and mucosae undergoing differentiation^5^. The integration of the high-risk HPV (hrHPV) DNA results in the constitutive expression of its oncogenes E6 and E7. Briefly, E6 oncoprotein binds to the cellular tumor suppressor protein p53 and directs its ubiquitin-mediated proteolytic degradation whereas E7 binds to and inactivates another cellular tumor suppressor protein Rb, thereby interfering the cell cycle control which leads to oncogenic growth^6–8^.

Although persistent hrHPV infection has become a well-established cause of cervical carcinogenesis, not all women infected with HPV develop cervical cancer, whereas women without HPV infection also develop cervical cancer^9^. This indicates the crucial role being played by variability in the host genetic factors, affecting women’s susceptibility to HPV infection and cervical cancer. One such factor is pathogen recognition receptors of the innate immune system, where Toll-like receptors (TLRs) have been identified as a key component playing a crucial role in the pathophysiology of varied human diseases, including cancer^10^.

TLRs are a part of innate immune system and significantly contribute in battling bacteria, viruses and other pathogens, and provide anti-tumor immunity^11^. TLRs serve as the initiator of inflammatory response generated by various factors including infection and tissue injury. Briefly, TLRs after binding to exogenous microbial or endogenous-tissue injury generated ligands activate transcription factors via adaptor protein myeloid differentiation factor 88 (MyD88) or MyD88 adaptor-like/Toll-interleukin 1 receptor domain–containing adaptor protein (Mal/TIRAP) leading to cytokines production and activation of adaptive immune response^12^.

To date, ten functional TLRs designated as TLR1 to TLR10 are expressed in humans by immune and certain non-immune cells. Of these TLRs, TLR1, 2, 4, 5, 6 and 10 are found on the cell surfaces whereas TLR3, 7, 8 and 9 are located in the endosomes or endoplasmic reticulum^13^. TLRs have also been implicated in the initiation, progression and metastasis of tumors^14,15^. Aberrant expression of different TLRs including TLR4 and TLR9 have been detected in gastric^16,17^, ovarian^18^, colorectal^19^, lung^20^, breast^21^, prostate^22^ as well as cervical cancers^23^. Furthermore, Hasan *et al*.^24^, reported the involvement of HPV16 E6 and E7 oncoproteins in the inhibition of TLR9 transcription, leading to decreased immune response and escape for HPV16.

Moreover, as inflammation is now considered as one of the crucial carcinogenic factors^12,25^, genetic variability in inflammation-associated *TLR* genes has revealed their potential role in influencing the susceptibility to pathogenic infections and development of cancer^10^. Of the various TLRs, TLR4 is known to recognise exogenous ligands such as lipopolysaccharide (LPS), fusion (F) protein of respiratory syncytial virus as well as endogenous ligand like heat shock proteins (HSP60, HSP70) and high mobility group box 1 (HMGB1)^26–28^, whereas TLR9 recognizes unmethylated CpG-rich bacterial and viral DNA^29^.

Reports on the influence of *TLR4* and *TLR9* single nucleotide polymorphisms (SNPs) in cervical cancer susceptibility are limited as well as conflicting^30–32^. In the case of *TLR4* polymorphisms, Asp299Gly (rs4986790) and Thr399Ile (rs4986791) were shown to be associated with tumor progression, however, no direct association of these SNPs was found in case-control set up^33,34^. Among the common *TLR9* polymorphisms -1486 T/C (rs187084) and C2848T (rs352140) polymorphisms were found to be the risk factors for cervical cancer^35–37^. Conversely, a study by Pandey *et al*.^38^ reported no association of *TLR9* C2848T polymorphism with cervical cancer, however, the same SNP was marginally associated with advanced cancer stages. Jin *et al*.^39^ reported a significant difference in the distribution of minor alleles of *TLR4* 3′ UTR SNP rs7873784 C/G and *TLR9* SNP G2848A in cervical cancer and HPV positive cases. However, in the same study group, the other *TLR4* SNPs (rs4986791, rs11536889) were not associated with cervical cancer.

Considering the importance of chronic inflammation in carcinogenesis as well as the influence of *TLR* genes’ polymorphisms in inflammation and cancer susceptibility, the present study was designed to investigate the role of four *TLR4* (rs4986790, rs10759931, rs11536889 and rs1927911) and equal number of *TLR9* (rs187084, rs5743836, rs352140 and rs352139) SNPs in HPV infection and cervical cancer susceptibility.

## Results

### Clinico-demographic characteristics

Mean age of cervical cancer patients (52.4 ± 11.6 years) and controls (51.8 ± 11.8 years) was comparable without any statistically significant difference (p = 0.625). However, features such as age at marriage (p < 0.001), age at first childbirth (p < 0.001) and parity (p < 0.0001) showed statistically significant difference between the cases and controls. All the cervical cancer cases were histopathologically diagnosed as squamous cell carcinoma type. Clinical staging of cervical cancer biopsies was performed as per the FIGO guidelines that revealed 9 (8.2%), 39 (35.5%), 55 (50%) and 7 (6.3%) patients in Stage I, II, III and IV respectively. The detailed demographic and clinicopathologic features of patients are presented in Table 1.

**Table 1:** Demographic features of cervical cancer cases and controls.

Variables	Cases	Controls	*P* value
**Age, year (mean** ± **SD)**	52.43 ± 11.68	51.8 ± 11.89	0.625
**Age at marriage, year (mean** ± **SD)**	18.22 ± 3.76	20.4 ± 4.37	<0.001
**Age at first child birth (mean** ± **SD)**	20.24 ± 4.58	22.83 ± 4.19	<0.001
**Parity**			
0–2	31 (28.1%)	106 (75.2%)	<0.0001
>2	79 (71.9%)	35 (24.8%)	
**FIGO classification**			
Stage I	9 (8.2%)		
Stage II	39 (35.5%)		
Stage III	55 (50%)		
Stage IV	7 (6.3%)		
**Histological types**			
Squamous cell carcinoma	110		
Adenocarcinoma	0		
Adenosquamous carcinoma	0		

Abbreviations: SD, standard deviation.

### HPV 16 and 18 prevalence

Prevalence of HPV as revealed by consensus primers in the cervical cancer cases was 81.6% (90/110), of which high-risk types 16 and 18 were detected in 64% (71/110) and 3.6% (4/110) cases. The combined frequency of HPV 16 and 18 was found to be 68% (75/110). Moreover, two out of 141 control subjects (1.4%) were also detected positive for HPV consensus sequences, of which one (0.7%) carried HPV16 DNA.

### Genotype distributions

All the *TLR4* and *TLR9* SNPs within the control population were in agreement with the Hardy-Weinberg equilibrium except for *TLR4* SNP rs11536889. However, the polymorphism was retained as its homozygous genotype GG was not detected in any of the study subjects which could be a probable reason for its deviation from the Hardy-Weinberg equilibrium.

A significant difference in the distribution of genotype frequencies between the cases and the controls were observed for *TLR4* SNPs rs11536889 (p = 0.013) and rs1927911 (p = 0.04) as well as *TLR9* SNPs rs187084 (p = 0.01) and rs352139 (p = 0.04). The distribution of genotypes for *TLR*4 and *TLR9* are shown in Supplementary Table S1. Association of *TLR4* and *TLR9* polymorphisms with HPV 16 and 18 infections is shown in Table 2. Individuals carrying heterozygous genotype of rs4986790 [p = 0.033, age-adjusted OR = 1.693 (1.043–2.747)], rs1927911 [p = 0.032, age-adjusted OR = 1.896 (1.055–3.406)] and rs187084 [p = 0.001, age-adjusted OR = 2.915 (1.508–5.635)] showed significant association with the presence of HPV 16 and 18 infections. Analysis of alleles among HPV 16/18 infected cases compared to controls revealed association of minor allele of rs4986790 [p = 0.031, age-adjusted OR = 1.789 (1.055–3.034)], rs1927911 [p = 0.023, age-adjusted OR = 1.653 (1.072–2.549)] and rs187084 [p = 0.036, age-adjusted OR = 1.538 (1.028–2.302)] SNPs with HPV 16/18 infection. Homozygous variant genotypes of rs1156889 [p = 0.013, age-adjusted OR = 1.948 (1.149–3.305)], rs187084 [p = 0.049, age-adjusted OR = 2.040 (1.009–4.126)] and heterozygous genotypes of rs1927911 [p = 0.003, age-adjusted OR = 2.248 (1.328–3.806)], rs187084 [p = 0.0002, age-adjusted OR = 3.004 (1.668–5.413)] were found to be associated with the increased risk of developing cervical cancer. Frequencies of the minor allele of *TLR4* SNPs rs4986790 [p = 0.033, age-adjusted OR = 1.693 (1.043–2.747)], rs1927911 [p = 0.013, age-adjusted OR = 1.635 (1.109–2.410)] and the major allele of *TLR9* SNP rs187084 were also varied significantly between patients and controls, conferring their association with the cervical cancer risk. Genotypic and allelic association between *TLR4* and *TLR9* variants and cervical cancer risk is presented in Table 3. A comparative analysis between early (stage I + II) and late (stage III + IV) stages revealed heterozygous genotypes of *TLR9* rs187084 [p = 0.011, age-adjusted OR = 0.283 (0.107–0.749)] and rs352140 [p = 0.015, age-adjusted OR = 0.304 (0.117–0.790)] to be associated with early stage cervical cancer. However, none of the *TLR4* SNPs shown significant association with early or late stages of cancer (Table 4).

**Table 2:** Genotypic association of *TLR4* and *TLR9* gene single nucleotide polymorphisms with HPV 16 and 18 infection.

Gene	SNP	Genotype/ Allele	Cases n (%)	OR^a^ (95% CI)	*P*^*a*^ value
*TLR4*	rs4986790	AA	46 (61.3)	1	
		AG	27 (36)	1.959 (1.056–3.635)	**0.033**
		GG	2 (2.7)	2.617 (0.346–19.795)	0.351
		A	119 (79.3)	1	
		G	31 (20.7)	1.789 (1.055–3.034)	**0.031**
	rs10759931	AA	13 (17.3)	1	
		AG	36 (48)	0.838 (0.380–1.847)	0.661
		GG	26 (34.7)	1.059 (0.458–2.448)	0.893
		A	62 (41.3)	1	
		G	88 (58.7)	1.065 (0.712–1.591)	0.760
	rs11536889	GG	NA		
		GC	47 (62.7)	1	
		CC	28 (37.3)	1.552 (0.854–2.820)	0.149
		G	47 (31.3)	1	
		C	103 (68.7)	1.238 (0.812–1.888)	0.322
	rs1927911	CC	28 (37.3)	1	
		CT	42 (56)	1.896 (1.055–3.406)	**0.032**
		TT	5 (6.7)	3.404 (0.852–13.601)	0.083
		C	98 (65.3)	1	
		T	52 (34.7)	1.653 (1.072–2.549)	**0.023**
*TLR9*	rs187084	TT	20 (28.1)	1	
		TC	40 (52.1)	2.915 (1.508–5.635)	**0.001**
		CC	15 (19.7)	1.793 (0.803–4.002)	0.154
		T	80 (53.3)	1	
		C	70 (46.7)	1.538 (1.028–2.302)	**0.036**
	rs5743836	TT	60 (81.7)	1	
		TC	13 (15.5)	0.562 (0.277–1.144)	0.112
		CC	2 (2.8)	1.724 (0.236–12.57)	0.586
		T	133 (88.7)	1	
		C	17 (11.3)	0.723 (0.395–1.322)	0.292
	rs352140	GG	21 (28.1)	1	
		GA	30 (40.8)	0.797 (0.403–1.575)	0.513
		AA	24 (30.1)	1.394 (0.659–2.950)	0.385
		G	72 (48)	1	
		A	78 (52)	1.186 (0.798–1.769)	0.395
	rs352139	AA	15 (18.3)	1	
		AG	49 (66.2)	1.589 (0.779–3.241)	0.203
		GG	11 (15.5)	0.610 (0.246–1.511)	0.285
		A	79 (52.7)	1	
		G	71 (47.3)	0.813 (0.547–1.209)	0.306

Abbreviations: SNP, single nucleotide polymorphism; OR, odds ratio; CI, confidence interval.

^a^Adjusted for age.

P value was calculated by a χ^2^–test and Fisher’s exact test using 2 × 2 contingency table (df = 1).

**Table 3:** Genotypic and allelic association of *TLR4* and *TLR9* single nucleotide polymorphisms with cervical cancer risk.

Gene	SNP	Genotype/ Allele	Cases n (%)	Controls n (%)	OR^a^ (95% CI)	*P*^*a*^ value
*TLR4*	rs4986790	AA	70 (63.6)	107 (75.9)	1	
		AG	37 (33.6)	32 (22.7)	1.767 (1.008–3.097)	0.0623
		GG	3 (2.7)	2 (1.4)	2.485 (0.397–15.56)	0.331
		A	177 (80.4)	246 (87.2)	1	
		G	43 (19.6)	36 (12.8)	1.693 (1.043–2.747)	**0.033**
	rs10759931	AA	18 (16.4)	23 (16.3)	1	
		AG	48 (43.6)	75 (54.2)	0.801 (0.390–1.644)	0.545
		GG	44 (40)	43 (30.5)	1.295 (0.613–2.735)	0.498
		A	84 (38.2)	121	1	
		G	136 (61.8)	161	1.150 (0.801–1.649)	0.449
	rs11536889	GC	63 (57.3)	102 (72.3)	1	
		CC	47 (42.7)	39 (27.7)	1.948 (1.149–3.305)	**0.013**
		G	63 (28.6)	102	1	
		C	157 (71.4)	180	1.385 (0.946–2.027)	0.094
	rs1927911	CC	37 (33.6)	76 (54.3)	1	
		CT	66 (60)	60 (42.9)	2.248 (1.328–3.806)	**0.003**
		TT	7 (6.4)	5 (2.9)	3.595 (0.989–13.064)	0.052
		C	140 (63.6)	212 (75.2)	1	
		T	80 (36.4)	70 (24.8)	1.635 (1.109–2.410)	**0.013**
*TLR9*	rs187084	TT	28 (25.5)	67 (47.5)	1	
		TC	58 (52.7)	46 (32.6)	3.004 (1.668–5.413)	**0.000**
		CC	24 (21.8)	28 (19.9)	2.040 (1.009–4.126)	**0.049**
		T	114 (51.8)	180 (63.8)	1	
		C	106 (48.2)	102 (36.2)	1.495 (1.042–2.143)	**0.029**
	rs5743836	TT	89 (80.9)	100(70.9)	1	
		TC	19 (17.3)	39 (27.7)	0.553 (0.297–1.029)	0.061
		CC	2 (1.8)	2 (1.4)	1.160 (0.159–8.474)	0.883
		T	197 (89.5)	239 (84.7)	1	
		C	23 (10.5)	43 (15.3)	0.704 (0.408–1.216)	0.208
	rs352140	GG	32 (29.1)	39 (27.7)	1	
		GA	45 (40.9)	70 (49.6)	0.782 (0.430–1.425)	0.422
		AA	33 (30)	32 (22.7)	1.255 (0.639–2.464)	0.510
		G	109 (49.5)	148 (52.5)	1	
		A	111 (50.5)	134 (47.5)	1.165 (0.818–1.660)	0.397
	rs352139	AA	23 (20.9)	33 (23.4)	1	
		AG	73 (66.4)	68 (48.2)	1.548 (0.826–2.900)	0.172
		GG	14 (12.7)	40 (28.4)	0.509 (0.226–1.147)	0.103
		A	119 (54)	134 (47.5)	1	
		G	101 (46)	148 (52.5)	0.759 (0.532–1.084)	0.129

Abbreviations: SNP, single nucleotide polymorphism; OR, odds ratio; CI, confidence interval.

^a^Adjusted for age.

P value was calculated by a χ^2^–test and Fisher’s exact test using 2 × 2 contingency table (df = 1).

**Table 4:** Genotypic association of *TLR4* and *TLR9* gene single nucleotide polymorphisms with early stage (I + II) and late stage (III + IV) of cervical cancer.

Gene	SNP	Genotype	Stage I + II n (%)	Stage III + IV n (%)	OR^a^ (95% CI)	*P*^*a*^ value
*TLR4*	rs4986790	AA	29 (60.4)	42 (67.7)	1	
		AG	16 (33.3)	20 (32.3)	0.870 (0.386–1.965)	0.738
		GG	3 (6.3)	0	0.333 (0.028–3.856)	0.564
		A	74 (77)	104 (83.8)	1	
		G	22 (23)	20 (16.2)	0.742 (0.336–1.638)	0.460
	rs10759931	AA	7 (14.6)	11 (17.7)	1	
		AG	20 (41.7)	28 (45.2)	0.855 (0.279–2.621)	0.783
		GG	21 (43.8)	23 (37.1)	0.680 (0.221–2.089)	0.501
		A	34 (35.4)	50 (40.3)	1	
		G	62 (64.6)	74 (59.7)	0.763 (0.353–1.647)	0.491
	rs11536889	GG	NA	NA		
		GC	28 (58.3)	35 (56.4)	1	
		CC	20 (41.7)	27 (43.6)	1.083 (0.505–2.324)	0.837
		G	28 (29.2)	35 (28.2)	1	
		C	68 (70.8)	89 (71.8)	1.083 (0.505–2.324)	0.837
	rs1927911	CC	15 (31.3)	21 (33.9)	1	
		CT	31 (64.6)	36 (58.1)	0.823 (0.363–1.869)	0.642
		TT	2 (4.2)	5 (8.1)	1.780 (0.303–10.45)	0.523
		C	61 (63.5)	78 (62.9)	1	
		T	35 (36.5)	46 (37.1)	0.881 (0.393–1.976)	0.759
*TLR9*	rs187084	TT	8 (16.7)	20 (32.2)	1	
		TC	34 (70.8)	24 (38.7)	0.283 (0.107–0.749)	**0.011**
		CC	6 (12.5)	18 (29.1)	1.194 (0.347–4.112)	0.779
		T	50 (52.1)	64 (51.6)	1	
		C	46 (47.9)	60 (48.4)	1.014 (0.595–1.730)	0.959
	rs5743836	TT	37 (77.1)	52 (83.9)	1	
		TC	11 (22.9)	8 (12.9)	0.509 (0.186–1.394)	0.189
		CC	0 (0)	2 (3.2)	4.091 (0.190–87.72)	0.745
		T	85 (88.5)	112 (90.3)	1	
		C	11 (11.5)	12 (9.7)	0.817 (0.343–1.944)	0.648
	rs352140	GG	10 (20.8)	22 (35.5)	1	
		GA	27 (56.3)	18 (29)	0.304 (0.117–0.790)	**0.015**
		AA	11 (22.9)	22 (35.5)	0.906 (0.320–2.567)	0.853
		G	47 (49)	62 (50)	1	
		A	49 (51)	62 (50)	1.027 (0.602–1.752)	0.921
	rs352139	AA	11 (22.9)	12 (19.3)	1	
		AG	31 (64.6)	42 (67.8)	1.498 (0.583–3.848)	0.401
		GG	6 (12.5)	8 (12.9)	2.044 (0.517–8.085)	0.308
		A	53 (55.2)	66 (53.2)	1	
		G	43 (44.8)	58 (46.8)	1.271 (0.742–2.177)	0.382

Abbreviations: SNP, single nucleotide polymorphism; OR, odds ratio; CI, confidence interval.

^a^Adjusted for age.

P value was calculated by a χ^2^–test and Fisher’s exact test using 2 × 2 contingency table (df = 1).

### Haplotype analysis

**Figure 1:**
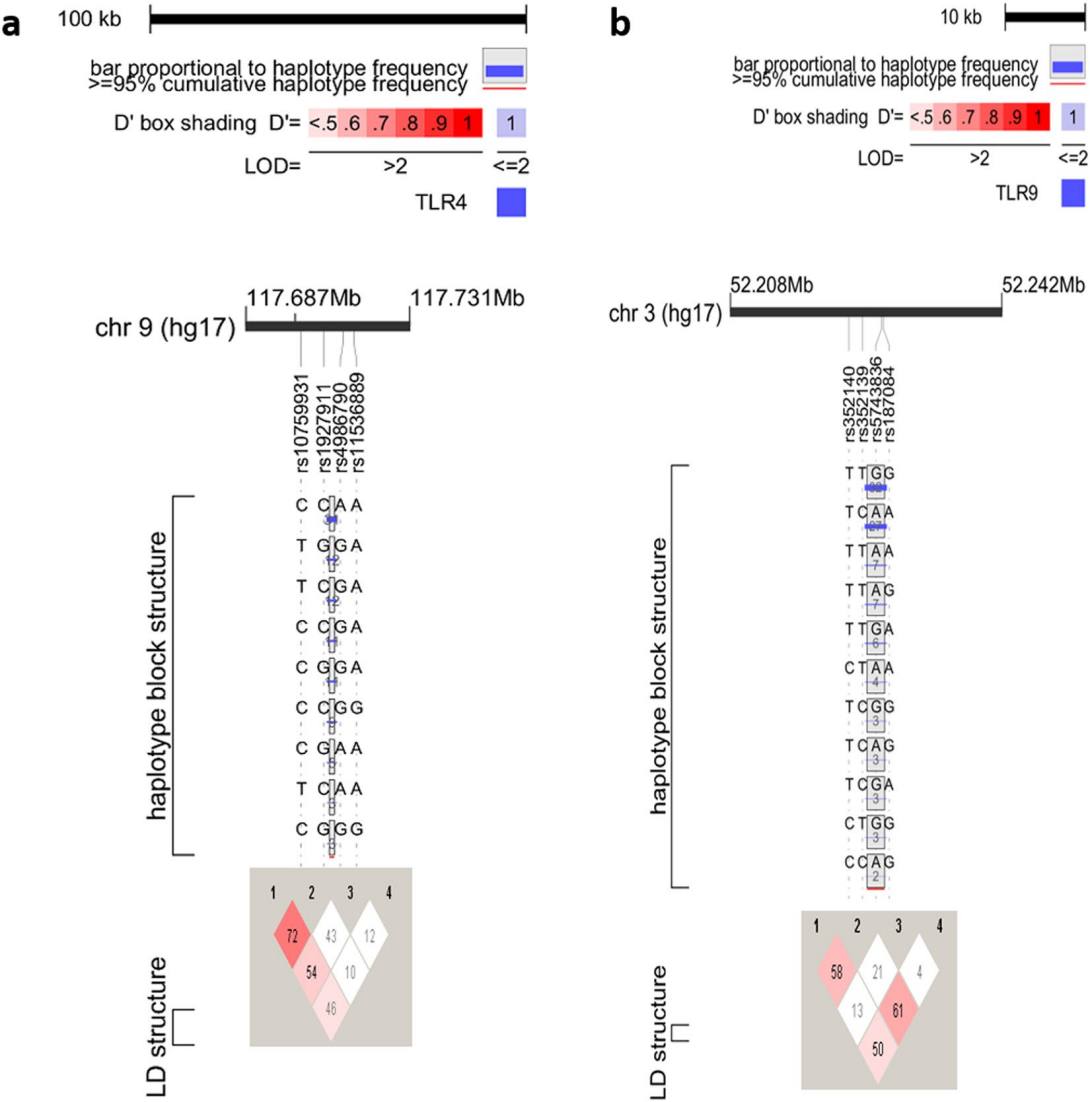
*TLR4* and *TLR9* haplotype block structures and linkage disequilibrium plots generated by Haploview and Locusview. (**a**) *TLR4* and (**b**) *TLR9* haplotype block structures, linkage disequilibrium plot and pairwise D′ value. The level of pair-wise D′ indicates the degree of linkage disequilibrium between two SNPs.

Linkage disequilibrium (LD) analysis revealed two SNPs of each *TLR4* (rs10759931 aka rs11536858, rs1927911) and *TLR9* (rs352139, rs187084) genes in strong LD (Fig. 1). The haplotypes were generated using the four SNPs of each *TLR4* and *TLR9* genes among the cases and controls (Table 5). Six common haplotype of *TLR4* (frequency > 5%) and *TLR9* (frequency > 2.5%) showed an accumulated frequency of 86.1% and 79.6% respectively in controls. Among HPV 16 and 18 positive patients *TLR4* and *TLR9* haplotypes revealed an accumulated frequency of 85.5% and 84.7% respectively. Distribution of *TLR4* haplotypes differed significantly in HPV 16 and 18 infected cases (Pglobal = 0.045) as compared to control, whereas no such difference was detected while evaluating the *TLR9* haplotypes (Pglobal = 0.493) (Table 6). *TLR4* haplotype GCAG [p = 0.0035, OR = 0.44 (0.20–0.96)] was associated with the decreased risk whereas *TLR9* haplotype GATC [p = 0.018, OR = 4.15 (1.16–14.80)] was associated with the increased risk of acquiring HPV 16 and 18 infection compared with controls.

**Table 5:** Association of *TLR4* and *TLR9* haplotypes with cervical cancer risk.

	Haplotype	Case Frequency (%)	Control frequency (%)	OR (95%CI)	*P* value
**Global P Value = 0.0033**					
*TLR4*	ACAC	28.4	32.2	0.82 (0.55–1.24)	0.395
	GTAG	14.2	11.2	1.32 (0.76–2.3)	0.336
	GTAC	15.9	9.7	1.77 (1.00–3.13)	**0.047**
	GCAC	8.7	12.4	0.67 (0.35–1.26)	0.22
	GCAG	5.7	13.6	0.39 (0.19–0.79)	**0.0076**
	GCGC	12.1	7	1.88 (1–3.51)	0.0628
**Global P Value = 0.227**					
*TLR9*	GGTT	28.4	34.5	0.75 (0.5–1.13)	0.171
	AATC	29.7	25.2	1.25 (0.82–1.90)	0.291
	AATT	7	6.5	1.09 (0.52–2.30)	0.816
	GATT	7	6.1	1.16 (0.54–2.45)	0.706
	AGTT	6	6	0.99 (0.45–2.18)	0.984
	GATC	5	1.3	3.95 (1.15–13.50)	**0.019**

Abbreviations: OR, odds ratio; CI, confidence interval.

**Table 6:** Association of *TLR4* and *TLR9* haplotypes with HPV 16 and 18 infection.

	Haplotype	Case Frequency (%)	Control frequency (%)	OR (95%CI)	*P* value
**Global P Value = 0.045**					
***TLR4***	ACAC	30	32.5	0.89 (0.56–1.39)	0.679
	GTAG	15.3	11.5	1.40 (0.77–2.55)	0.279
	GCAG	6.4	13.5	0.44 (0.20–0.96)	**0.035**
	GTAC	14.6	9.6	1.62 (0.86–3.04)	0.135
	GCAC	7.6	12.3	0.58 (0.28–1.22)	0.155
	GCGC	11.6	7.1	1.72 (0.86–3.44)	0.128
**Global P Value = 0.493**					
***TLR9***	GGTT	31.5	34.6	0.87 (0.56–1.35)	0.528
	AATC	29.8	25.4	1.25 (0.79–1.98)	0.343
	AATT	6.0	6.4	0.93 (0.39–2.22)	0.869
	AGTT	6.6	6.0	1.11 (0.48–2.60)	0.805
	GATT	5.4	6.1	0.88 (0.35–2.17)	0.775
	GATC	5.4	1.3	4.15 (1.16–14.80)	**0.018**

Abbreviations: OR, odds ratio; CI, confidence interval.

Among cervical cancer cases, *TLR4* and *TLR9* haplotypes revealed an accumulated frequency of 85% and 83.1% respectively. Results of the global test score showed a significant difference in haplotype distribution between patients and controls in the case of *TLR4* variants (Pglobal = 0.0033), while no significant difference was obtained for *TLR9* variants (Pglobal = 0.227) (Table 5). Furthermore, the *TLR4* haplotype GTAC [p = 0.047, OR = 1.77 (1.00–3.13)] and *TLR*9 haplotype GATC [p = 0.019, OR = 3.95 (1.15–13.50)] were found to be associated with the increased risk of cervical cancer whereas the *TLR4* haplotype GCAG [p = 0.0076, OR = 0.39 (0.19–0.79)] was significantly associated with decreased risk of cervical cancer. Furthermore, within cases, haplotypes analysis did not reveal an association of either *TLR4* (Pglobal = 0.733) or *TLR9* (Pglobal = 0.546) haplotypes with the early or late stages of cervical cancer (Supplementary Table S2).

## Discussion

The influence of *TLR* polymorphisms is gradually increasing in the field of biomarkers study in various diseases including cancer^10^. In the present study, we investigated the role of the common *TLR4* and *TLR9* SNPs in susceptibility to HPV infection and cervical cancer among the study subjects from Gujarat, India. Considering the influence of hrHPVs in cervical carcinogenesis, we first analyzed the prevalence of two major hrHPVs HPV 16 and 18 that revealed a frequency of 68% as compared to nearly 71% and 78% prevalence globally as well as in India respectively^40^. However, a previous report^41^, from the same geographic region as of ours found 60% of the patients to be infected with HPV 16 and 18. The difference in the percentage of hrHPV detection, though not very high, can be attributed to the variation in the sample size as the number of patients in the present study were more than double as reported by Patel *et al*.^41^. A higher prevalence of approximately 21% HPV infection other than HPV 16 and 18 in our study subjects highlights the necessity of genotyping other hrHPVs to identify additional prevailing HPVs.

We further analyzed polymorphisms present in UTRs, exons, and introns of *TLR4* and *TLR9* genes. The variations in UTRs are known to influence ribosome recognition, termination and post-transcriptional modification which may alter the expression and functionality of a particular protein^42^. We found a mixed association of different 3′ UTR and 5′ UTR SNPs of *TLR4* and *TLR9* genes in our study subjects, suggesting a probable role of these SNPs in disease susceptibility.

*TLR9* promoter SNP rs187084 (-1486T/C) conferred a increased risk to HPV 16 and 18 infection and cervical cancer. A similar association of *TLR9* rs187084 polymorphism with an increased risk of cervical cancer was reported among Polish and Chinese women^35,36^. Our results on *TLR9* rs187084 polymorphism are in good agreement with the recent meta-analyses^30,31^ that supported a significant role of rs187084 in cervical cancer risk. Within cases, *TLR9* rs187084 showed over presentation in early-stage cancer compared with late stages. Interestingly, we did not find an association of another *TLR9* promoter SNP rs5743836 (−1237T/C) with HPV infection and/ or cervical cancer risk. Our result supports the observation of Oliveira *et al*.^43^ who reported no association of *TLR9* promoter SNP rs5743836 with HPV infection or clearance in healthy Brazilian women. Even though no direct role of *TLR9* promoter SNPs has been reported in cervical cancer, the T allele of *TLR9* promoter SNP rs187084 (−1486T/C) together with G allele of intronic rs352139 A/G SNP have been suggested to down regulate *TLR9* expression in systemic lupus erythematosus^44^. The T allele of rs5743836 (−1237T/C) has been suggested to be associated with high basal promoter activity^45^ and C allele with higher affinity to NF-κB binding, causing increased production of proinflammatory cytokines^46^.

With regard to *TLR4* promoter SNP rs10759931, no association was observed either with HPV infection or cervical cancer risk. However, the same SNP has been reported to be associated with prostate and gastric cancers risk^47,48^. The homozygous AA genotype of *TLR4* rs10759931 has been reported to be associated with high *TLR4* expression in symptomatic atherosclerotic patients compared to non-symptomatic and healthy individuals carrying GG or GA genotypes^49^. They found that the two alleles of rs10759931 differ in their binding affinity to GATA-2 transcriptional factor. Furthermore, we observed the 3′ UTR heterozygous genotype GC of *TLR4* rs11536889 to be associated with increased risk of cervical cancer in our study subjects. A similar observation was found in bladder cancer^50^, however, the association status of this SNP with other cancers was inconsistent^32^. Moreover, the G allele of *TLR4* rs11536889 3′ UTR SNP has been suggested to play a key role in inhibiting *TLR4* translation in monocytes^51^. However, expression analysis of *TLR4* and *TLR9* genes may provide more insights into the functional role of these UTR SNPs in cervical cancer risk.

Additionally, we analyzed a synonymous and a non-synonymous SNP of *TLR9* and *TLR4* genes respectively. Even though a synonymous change does not alter incorporation of amino acid, it has been observed that such SNPs can alter mRNA splicing, stability, and structure as well as protein folding thereby affecting the function of the subsequent protein^52^. We did not find a significant effect of *TLR9* synonymous SNP rs352140 (G2848A; Pro545Pro) with cervical cancer risk which is in good agreement with a recent meta-analysis by Tian *et al*.^30^. An association of G2848A SNP with early stages of cervical cancer was detected in our study subjects which is in contrast to the report of Pandey *et al*.^38^ who observed an association of the same SNP with the late stage cervical cancer in North Indian women. However, Roszak *et al*.^35^ reported an association of C2848T SNP along with –1486T/C SNP with cervical cancer risk in the Polish population. Similarly, the Han Chinese women carrying *TLR9* rs352140 (G2848A) GA/AA genotype along with HPV16 infection showed an increased risk of cervical cancer compared to women with GG genotype^35,53^.

With regard to non-synonymous SNP rs4986790 (A896G; Asp299Gly) of *TLR4*, intriguingly, we found the heterozygous AG genotype (Asp/Gly) to be strongly linked to HPV 16/18 infection suggesting a queering effect of the amino acid change as no interaction of HPV capsid proteins with TLR4 is known yet. The amino acid change is reported to affect van der Waals interaction and hydrogen bonding in the leucine-rich repeats of TLR4, thereby modulating its surface properties that may affect the binding of TLR4 ligand such as LPS^54^. Although HPV is not a known TLR4 ligand, our paradoxical observation warrants a meticulous investigation. Furthermore, we observed a significant association of minor allele G (Gly) of Asp299Gly polymorphism with cervical cancer risk, however, no genotypic association was found. Similarly, in North Indian women, no association of *TLR4* Asp299Gly polymorphism, in addition to another common *TLR4* Thr399Ile polymorphism with cervical cancer risk was observed by Pandey *et al*.^33^. Moreover, Asp299Gly polymorphism has been found to be contradictorily associated with different cancer types including cervical cancer^32^.

A growing body of evidence suggests a potential role of intronic SNPs located either in exon/ intron boundaries, intron splice enhancer, branchpoint site or outside the exon-intron splice junctions in regulating gene expression^55^. It has also been observed that intronic SNPs in one gene can affect the expression of a far located gene^55^. Congruously, we observed a significant difference in the distribution of genotypes of *TLR9* intronic rs352139 A/G SNP between cases and controls, however, none of its genotypes or allele was associated with cervical cancer risk. On the other hand, the heterozygous genotype of *TLR4* intronic rs1927911 SNP was significantly associated with cervical cancer risk which is in agreement with the observation of Song *et al*.^47^ in prostate cancer. However, in hepatocellular carcinoma, the same SNP showed a protective effect^56^.

As haplotypes are considered more informative than SNPs^57^, we generated haplotypes from different combinations of *TLR4* and *TLR9* SNPs. The *TLR4* haplotype GTAC was linked with a significant increase in cervical cancer risk in addition to the *TLR9* haplotype GATC that also showed association with increased HPV 16 and 18 infections. Intriguingly, another *TLR4* haplotype GCAG showed a significant association with decreased cervical cancer risk as well as acquiring the hrHPV infection, suggesting its protective role. Moreover, to understand the influence of *TLR4* and *TLR9* haplotypes on tumor progression, we correlated the haplotypes with early (I and II) and late (III and IV) tumor stages. However, none of the haplotypes showed association with clinical aggressiveness. Since these haplotypes included both risk as well as protective alleles, a crucial role of *TLR4* and *TLR9* polymorphisms may be envisaged towards HPV infection and cervical cancer susceptibility.

To identify the strong coinheritance of the SNPs we calculated linkage disequilibrium of *TLR4* and *TLR9* SNPs, wherein *TLR4* rs10759931 and rs1927911, and *TLR9* rs187084 and rs352139 were in strong LD, evincing strong influence of these inherited variations in cervical cancer. Intriguingly, we observed that in both the genes strong LD was detected between SNPs of 5′ UTR and the first intron only. Conceptually there should be a decrease in linkage disequilibrium with a decrease in distance between two loci. However, our study revealed SNP pairs in both *TLR4* and *TLR9* genes that did not follow the standard notion. For example, in *TLR4*, SNP pair rs10759931:rs4986790 with a distance of 11.1 Kb showed strong LD (D′ = 0.54) as compared to another SNP pair rs4986790:rs11536889 that had a shorter distance of 2.8Kb (D′ = 0.12). Similarly, *TLR9* SNP pair rs352140:rs187084 (distance = 4.3 kb) was in strong LD (D′ = 0.5) compared to SNP pair rs5743836:rs187084 (D′ = 0.04) with shorter distance of 0.24 kb among them. Our LD analysis is in agreement with the observations of Stephens *et al*.^57^ who suggested that distance between the SNPs does not have a significant impact on the level of LD. Various SNP pairs of *TLR4* and *TLR9* genes, their genetic distance and D′ values are shown in Supplementary Table S3.

Although our results suggest a significant role of *TLR4* and *TLR9* polymorphisms in cervical cancer, the study has some vital limitations too. Firstly, the selection bias cannot be excluded as it was a hospital-based case-control study, Moreover, the size of the study population needed augmentation to increase the statistical power, which is one of the major limiting factors among the numerous cancer case-controls studies worldwide. Additionally, *in vivo* expression analysis would have reflected the effect of SNPs on the expression pattern of TLR4 and TLR9.

To our knowledge, this is the first comprehensive analysis of *TLR4* and *TLR9* SNPs and haplotypes to understand their role in cervical cancer. Our results suggest moderate to strong impact of *TLR4* and *TLR9* polymorphisms in susceptibility to hrHPV infection and cervical cancer. Additional research on large and varied ethnic populations is warranted to precisely understand the impact of both the genes in HPV infection and cervical cancer risk.

## Methods

### Study subjects

The study comprised of 110 untreated cervical cancer patients and 141 healthy controls recruited from 2012 to 2017; from Shree Krishna Hospital, Karamsad, Anand; and Sir Sayajirao General Hospital and Medical College, Vadodara, India. The sample types included primary histopathologically diagnosed cervical cancer biopsies and cytologically confirmed normal cervical smears from healthy controls. The clinical staging of cervical cancer samples was done as per The International Federation of Gynecology and Obstetrics (FIGO) recommendations. The study subjects belonging to Gujarati ethnicity were comparable in age and non-relatives of each other. The patients manifesting multiple cancers and those who underwent radiation or chemotherapy were excluded from the study. The inclusion criteria of healthy controls included the absence of cancer history in family and cervix related disorders such as cervicitis, warts, pre-cancerous and cancerous lesions. Additionally, sample collection was avoided from the women undergoing menstruation. All experiments were performed in accordance with the relevant guidelines and regulations. The study was approved by the Institutional Review Board, Ashok and Rita Patel Institute of Physiotherapy, CHARUSAT, Changa, Anand; Institutional Ethics Committee, HP Patel Centre for Medical Care and Education, Karamsad and Institutional Ethics Committee for Human Research (IECHR) Medical College and SSG Hospital, Vadodara, India. Written informed consent was obtained from all the study subjects.

### DNA extraction

The samples were collected in chilled phosphate buffered saline and were either processed immediately or stored at −20 °C till further processing. DNA was isolated using standard Proteinase-K phenol-chloroform extraction method. In the case of a low number of cervical cells, spin-column based DNA isolation kit (NucleoSpin Tissue, Macherey-Nagel, Germany) was utilized. The quality and quantity of extracted DNA were determined using ethidium bromide-stained 1% agarose gel on a GelDoc system (BioRad, USA) and NanoDrop 2000 (Thermofisher, USA).

### HPV detection

HPV detection was first carried out using consensus Gp5+/Gp6+ primers followed by type-specific primers for the detection of hrHPV 16 and 18, on a Real-time PCR platform (7500 Real-Time PCR System, Applied Biosystems, USA) using SYBR Premix Ex Taq II (Tli RNaseH Plus) kit (Takara, Japan). Typically, a 20 μl real-time PCR mix comprised of 1X SYBR Premix Ex Taq (Tli RNAse H Plus), 0.2 µM of each forward primer and reverse primer, 1X ROX reference Dye II and 25 ng of template DNA. The positive controls for HPV 16 and 18 were obtained as a part of participation in the Global HPV Proficiency Study, Equalis, Uppsala, Sweden. β-globin gene served as an internal control while in the negative control DNA was replaced with PCR grade nuclease-free water. All the reactions were performed in duplicates. Touchdown thermal profile for HPV detection by consensus primers and thermal cycling conditions for HPV 16 and 18 detections along with the details of primer sequence and amplicon size is mentioned in Supplementary Table S4.

### Genotype analyses

A total of eight SNPs, four each of *TLR4* (rs4986790, rs10759931, rs11536889, rs1927911) and *TLR9* (rs187084, rs5743836, rs352140, rs352139) genes were analyzed either using Polymerase Chain Reaction and Restriction Fragment Length Polymorphism (PCR-RFLP) or Allele-Specific PCR (AS-PCR). The selection of SNPs was carried out using SNP database of NCBI (https://www.ncbi.nlm.nih.gov/snp/). The SNPs were selected on the basis of (1) Genetic region: In this criteria the SNPs were selected to cover different regions of gene, for example, exon, intron and UTRs, (2) Global minor allele frequency: The SNPs with minor allele frequency > 5% were evaluated for association analysis (3) Frequent association of SNPs with different inflammation associated cancers: To fulfil the above criteria literature survey was conducted using PubMed and random web search. The characteristics of *TLR4* and *TLR9* SNPs included in this study are shown in Supplementary Table S5. Sequences of primers specific for each SNP, amplicon size and thermal profile is mentioned in Supplementary Table S6. A typical PCR of 25 µl contained 50 to 100 ng genomic DNA, 0.1 mM dNTP mix, 0.1 µM of each oligonucleotide primer and 0.8U Taq DNA polymerase (Kapabiosystems, USA). All the reactions were performed on an MJ Mini Thermal Cycler (BioRad, USA). Except for *TLR9* rs352139 polymorphism that was genotyped using AS-PCR, the rest of the SNPs were subjected to restriction digestion using 5U of respective restriction enzymes procured from New England Biolabs, USA. For the identification of SNPs by RFLP, the associated restriction enzymes, incubation temperature and time, digested products, genotypes and mode of visualization is detailed in Supplementary Table S7. The amplified, as well as restriction digested products, were visualized on a GelDoc system (BioRad, USA).

### Statistical analysis

Alterations in demographic features among cases and controls were compared using student t-test and chi-square test for continuous and categorical variables respectively. Age of study subjects was expressed as mean ± standard deviation. Deviation from Hardy–Weinberg equilibrium was determined by the χ^2^ goodness-of-fit test. Pearson’s χ^2^ test was used to evaluate the difference of the SNP distribution among cases and controls. Genotypic and allelic association of SNPs with the disease were estimated using χ^2^ and Fisher’s exact test. Unconditional logistic regression analysis was performed to compute age-adjusted odds ratio (OR). All the statistical analysis was performed on the Statistical Package for Social Sciences version 24.0 (SPSS, USA). Tests of statistical significance were two-sided and taken as significant when the p-value was less than 0.05. Haplotype block structure and linkage disequilibrium (LD) structure were determined by Haploview (v4.2) and Locusview (v2.0). The D′ values were computed using the default algorithm created by Gabriel *et al*.^58^ at 95% confidence interval. Haplotypes were estimated using an accelerated EM algorithm similar to the partition/ ligation method as described by Qin *et al*.^59^. Sum of the fractional likelihoods of each individual for each haplotype was used to obtain a count for case-control association tests. Global score test was performed using FAMHAP software v19 to evaluate the differences in haplotype frequency distribution among cases and controls. Association of the individual haplotype with cervical cancer as well as HPV infection was measured by the χ^2^ test.

## Supplementary information

Electronic supplementary material

## Data Availability

All data generated or analysed during this study are included in this published article (and its Supplementary Information files).
