# Author Correction: Association of *TLR4* and *TLR9* polymorphisms and haplotypes with cervical cancer susceptibility

**DOI:** 10.1038/s41598-019-55122-w

**Published:** 2019-12-04

**Authors:** Nilesh O. Pandey, Alex V. Chauhan, Nitin S. Raithatha, Purvi K. Patel, Ronak Khandelwal, Ajesh N. Desai, Yesha Choxi, Rutul S. Kapadia, Neeraj D. Jain

**Affiliations:** 1 P D Patel Institute of Applied Sciences, Charotar University of Science and Technology (CHARUSAT), Changa, Anand, India; 2 Department of Obstetrics and Gynaecology, Pramukh Swami Medical College, Shree Krishna Hospital, Karamsad, Anand, India; 3 Department of Obstetrics and Gynaecology, Sir Sayajirao General Hospital and Medical College, Vadodara, India; 4 Department of Obstetrics & Gynaecology, GMERS Medical College and Hospital, Ahmedabad, India

Correction to: *Scientific Reports* 10.1038/s41598-019-46077-z, published online 05 July 2019

The Author Contributions section in this Article is incorrect.

“All the authors have contributed significantly, read the manuscript, and agrees to its submission to Gynecologic Oncology. In this study N.P. and A.C. have performed experimental work, analysis and interpretation of data, preparation and drafting of manuscript. N.R., P.P. and A.D. have supervised the study as clinical investigators and critically reviewed the study proposal. R.K., Y.C. and R.S.K. have contributed in sample/data collection and analysis, and reviewed the manuscript critically. The task of conceptualization, funding acquisition, project administration, supervision, validation, review and editing was performed by N.J.”

should read:

“All the authors have contributed significantly, read the manuscript, and agrees to its submission to Scientific Reports. In this study N.P. and A.C. have performed experimental work, analysis and interpretation of data, preparation and drafting of manuscript. N.R., P.P. and A.D. have supervised the study as clinical investigators and critically reviewed the study proposal. R.K., Y.C. and R.S.K. have contributed in sample/data collection and analysis, and reviewed the manuscript critically. The task of conceptualization, funding acquisition, project administration, supervision, validation, review and editing was performed by N.J.”

