# Supplementary material for: Association of TLR4 and TLR9 polymorphisms and haplotypes with cervical cancer susceptibility
Source: Sci Rep. 2019 Jul 5;9:9729. doi: 10.1038/s41598-019-46077-z (PMC6611874; doi:10.1038/s41598-019-46077-z)
Supplement: Supplementary file 1 — Electronic supplementary material [file 41598_2019_46077_MOESM1_ESM.pdf]

Association of *TLR4* and *TLR9* polymorphisms and haplotypes with cervical cancer susceptibility

Nilesh O. Pandey[1#], Alex V. Chauhan[1#], Nitin S. Raithatha[2], Purvi K. Patel[3], Ronak Khandelwal[3], Ajesh N. Desai[4], Yesha Choxi[4], Rutul S. Kapadia[4], and Neeraj D. Jain[1*]

[1] P D Patel Institute of Applied Sciences, Charotar University of Science and Technology, Changa, India.

[2] Department of Obstetrics and Gynaecology, Pramukh Swami Medical College, Shree Krishna Hospital, Karamsad, India.

[3] Department of Obstetrics and Gynaecology, Sir Sayajirao General Hospital and Medical College, Vadodara, India.

[4] Department of Obstetrics & Gynaecology, GMERS Medical College and Hospital, Ahmedabad, India.

Table S1: Comparison of genotypic frequencies of *TLR4* and *TLR9* single nucleotide polymorphisms between cases and controls

Gene	SNP	Genotype	Cases n (%)	Controls n (%)	P value
TLR4	rs4986790	AA	70 (63.6)	107 (75.9)	0.103
		AG	37 (33.6)	32 (22.7)	
		GG	3 (2.7)	2 (1.4)	
	rs10759931	AA	18 (16.4)	23 (16.3)	0.251
		AG	48 (43.6)	75 (54.2)	
		GG	44 (40)	43 (30.5)	
	rs11536889	GG	0 (0)	0 (0)	**0.013**
		GC	63 (57.3)	102 (72.3)	
		CC	47 (42.7)	39 (27.7)	
	rs1927911	CC	37 (33.6)	76 (54.3)	**0.04**
		CT	66 (60)	60 (42.9)	
		TT	7 (6.4)	4 (2.9)	
TLR9	rs187084	TT	28 (25.5)	67 (47.5)	**0.01**
		TC	58 (52.7)	46 (32.6)	
		CC	24 (21.8)	28 (19.9)	
	rs5743836	TT	89 (80.9)	100 (70.9)	0.152
		TC	19 (17.3)	39 (27.7)	
		CC	2 (1.8)	2 (1.4)	
	rs352140	GG	32 (29.1)	39 (27.7)	0.309
		GA	45(40.9)	7 (49.6)	
		AA	33 (30)	32 (22.7)	
	rs352139	AA	23 (20.9)	33 (23.4)	**0.04**
		AG	73 (66.4)	68 (48.2)	
		GG	14 (12.7)	40 (28.4)	

Abbreviations: SNP, Single nucleotide polymorphism
P value was calculated by a $\chi2$–test 3X2 contingency table (df = 2)

Table S2: Association of *TLR4* and *TLR9* haplotypes with early and late stages of cervical cancer

	Haplotype	Stage III+IV (%)	Stage I+II (%)	OR (95%CI)	P value
		Global P Value= 0.733			
TLR4	ACAC	35.9	30	1.26 (0.61-2.57)	0.459
	GTAG	15.4	12.8	1.28 (0.49-3.35)	0.652
	GTAC	11.5	12.4	0.88 (0.31-2.46)	0.871
	GCGC	10.7	6.9	1.75 (0.54-5.59)	0.431
	GCAC	6.6	12.5	0.49 (0.15-1.6)	0.222
	GCGG	5.0	9.6	0.37 (0.08 - 1.62)	0.287
		Global P Value= 0.546			
	**Haplotype**	**Stage III+IV (%)**	**Stage I+II (%)**	**OR (95%CI)**	**P value**
TLR9	AATC	25.6	34.6	0.65 (0.31-1.35)	0.245
	GGTT	23.1	31.9	0.64 (0.3-1.35)	0.238
	AATT	10.3	6.4	1.69 (0.48-5.91)	0.409
	AGTT	7.1	5.9	1.2 (0.31-4.7)	0.79
	GATT	7.0	4.4	1.66 (0.37-7.45)	0.505
	GATC	5.1	6.3	0.79 (0.19-3.32)	0.751

Abbreviations: OR, odds ratio; CI, Confidence Interval

Table S3: The SNP pairs, genetic distance and D' values of *TLR4* and *TLR9* gene polymorphisms.

Gene	SNP pairs	Distance (bp)	LD (D′)
TLR4	rs10759931-rs1927911	5907	0.72
	rs10759931-rs4986790	11155	0.54
	rs10759931-rs11536889	13984	0.46
	rs1927911-rs4986790	5248	0.43
	rs1927911-rs11536889	8077	0.1
	rs4986790 -rs11536889	2829	0.12
TLR9	rs352140-rs352139	1675	0.58
	rs352140-rs5743836	4085	0.13
	rs352140-rs187084	4334	0.5
	rs352139-rs5743836	2410	0.21
	rs352139-rs187084	2659	0.61
	rs5743836-rs187084	249	0.04

Abbreviations: SNP, single nucleotide polymorphisms; bp, base-pairs. LD, linkage disequilibrium

Table S4: Primer sequence, thermal conditions and amplicon size for HPV detection

Primer name	Sequence (5'-3')	Thermal Condition	Amplicon size (bp)	Reference
Gp 5+	TTTGTTACTGTGGTAGATACTAC	(95°-1')1 (95°-20", 55°C to 40°C with 1.0°C decrements -30", 72°-30")16 (95°-20", 40°-30", 72°-30")34 (72°-4')1	150	[1]
Gp 6+	GAAAAATAAACTGTAAATCATATTC			
HPV 16 FP	AAGGCCAACTAAATGTCAC	(95°-1')1 (95°-15", 55°-30", 72°-30")40 (72°-4')1	216	[2]
HPV 16 RP	CTGCTTTTATACTAACCGG			
HPV 18 FP	ACCTTAATGAAAAACCACGA	(95°-1')1 (95°-15", 55°-30", 72°-30")40 (72°-4')1	100	
HPV 18 RP	CGTCGTTTAGAGTCGTTCCTG			

Abbreviations: FP, forward primer; RP, reverse primer

Table S5: *TLR4* and *TLR9* SNPs characteristics

Gene	SNP	rs ID	Location	Nucleotide Change	Amino acid Change	Global MAF
TLR4	A896G	rs4986790	Exon	A→G	Asp→Gly	5.9
	A2688G	rs10759931	5' UTR	A→G	NA	35.1
	G3725C	rs11536889	3' UTR	G→C	NA	13.7
	C7764T	rs1927911	Intron	C→T	NA	40.0
TLR9	T-1486C	rs187084	5' UTR	T→C	NA	37.7
	T-1237C	rs5743836	5' UTR	T→C	NA	17.2
	G2848A	rs352140	Exon	G→A	Pro→Pro	41.5
	A1174G	rs352139	Intron	A→G	NA	49.0

Abbreviations: SNP, single nucleotide polymorphism; MAF, minor allele frequency; UTR, untranslated region

Table S6: Primer sequence, thermal conditions and amplicon size for genotyping of *TLR4* and *TLR9* gene polymorphism

Gene	rsID	Primer (5'-3')	Thermal condition	Amplicon Size (bp)
TLR4	rs4986790	F: GATTAGCATACTTAGACTACTACCTCCATG<br>R: GATCAACTTCTGAAAAAGCATTCCCAC	(95°-5')1 (94°-40", 55°-40", 72°-60")36 (72°-10')1	249
	rs10759931	F: ATAACCTCAGTGGGCTCTGG<br>R: ATGTTCTGGCATCTGGGAAG	(94°-5')1 (94°-40", 58°-45", 72°-40")35 (72°-10')1	241
	rs11536889	F: ACAAGTGATGTTTGATGGAC<br>R: GCCATTCTACCTGGTATAAG	(94°-6')1 (94°-60", 55°-60", 72°-2’)35 (72°-10')1	361

	rs1927911	F: TCACTTTGCTCAAGGGTCAA<br>R: AAACCTGCATGCTCTGCAC	(94°-5')1 (94°-40", 58°-45", 72°-40")35 (72°-10')1	203
TLR9	rs187084	F: TCCCAGCAGCAACAATTCATTA<br>R: CTGCTTGCAGTTGACTGTGT	(95°-5')1 (95°-40", 60°-40", 72°-60")36 (72°-10')1	499
	rs5743836	F: ATGGGAGCAGAGACATAATGGA<br>R: CTGCTTGCACTTGACTGTGT	(95°-5')1 (94°-40", 62°-40", 72°-60")35 (72°-10')1	135
	rs352140	F: AAGCTGGACCTCTACCACGA<br>R: TTGGCTGTGGATGTTGTT	(95°-5')1 (94°-45", 56°-60", 72°-30")35 (72°-10')1	177
	rs352139	AFP: AAGTGGAGTGGGTGGAGGTA<br>GFP: GTGGAGTGGGTGGAGGTG<br>R: CAAGGAAAGGCTGGTGACAT	(95°-5')1 (94°-60", 64°-60", 72°-60")35 (72°-4')1	270

Abbreviations: F, forward primer; R, reverse primer

Table S7: Information regarding restriction enzymes, digested products and interpretation of genotypes for different *TLR4* and *TLR9* SNPs.

Gene	rsID	Genotyping method	Restriction enzyme	Incubation temperature (°C)	Digested product (bp) / PCR product (bp)	Separation	Reference
TLR4	rs4986790	PCR-RFLP	NcoI	37	AA: 249<br>AG: 249, 223, 26<br>GG: 223, 26<br>CT: 406, 377, 29<br>TT: 377, 29	15% PAGE	[3]
	rs10759931	PCR-RFLP	KpnI	37	AA: 241<br>AG: 241, 190, 51<br>GG: 190, 51	2.5% Agarose	[4]
	rs11536889	PCR-RFLP	EarI	37	GG: 198, 163<br>GC: 361, 198, 163<br>CC: 361	2.5% Agarose	[5]
	rs1927911	PCR-RFLP	StyI	37	CC: 203<br>CT: 203, 178, 25<br>TT: 178, 25	15% PAGE	[4]
TLR9	rs187084	PCR-RFLP	AflII	37	TT: 327, 172<br>TC: 499, 327, 172<br>CC: 499	2% Agarose	[3]
	rs5743836	PCR-RFLP	BstNI	60	TT: 108, 27<br>TC: 108, 60, 48, 27<br>CC: 60, 48, 27	15% PAGE	[3]
	rs352140	PCR-RFLP	BstUI	60	GG: 177<br>GA: 177, 135, 42<br>AA: 135, 42	12% PAGE	[6]
	rs352139	AS- PCR	NA	NA	NA	2% Agarose	[7]

Abbreviations: PCR-RFLP, polymerase chain reaction- restriction fragment length polymorphism; AS-PCR, Allele specific PCR; PAGE, polyacrylamide gel electrophoresis

References

1. Evans, M. F., Adamson, C. S., Simmons-Arnold, L. & Cooper, K. Touchdown General Primer (GP5+/GP6+) PCR and optimized sample DNA concentration support the sensitive detection of human papillomavirus. *BMC Clin. Pathol.* **5,** 10 (2005).

2. Jain, N. *et al.* Infection of human papillomavirus type 18 and p53 codon 72 polymorphism in lung cancer patients from India. *Chest* **128,** 3999–4007 (2005).

3. Liu, F. *et al.* Frequency of TLR 2 , 4 , and 9 Gene Polymorphisms in Chinese Population and Their Susceptibility to Type 2 Diabetes and Coronary Artery Disease. *J Biomed Biotechnol* **2012,** (2012).

4. Singh, K., Singh, V. K., Agrawal, N. K., Gupta, S. K. & Singh, K. Association of Toll-Like Receptor 4 Polymorphisms with Diabetic Foot Ulcers and Application of Artificial Neural Network in DFU Risk Assessment in Type 2 Diabetes Patients. *Biomed Res Int* **2013,** (2013).

5. Shen, Y., Liu, Y., Liu, S. & Zhang, A. Toll-like receptor 4 gene polymorphisms and susceptibility to bladder cancer. *Pathol. Oncol. Res.* **19,** 275–80 (2013).

6. Pandey, S., Mittal, B. & Srivastava, M. Evaluation of Toll-like receptors 3 (c . 1377C / T) and 9 (G2848A) gene polymorphisms in cervical cancer susceptibility. *Mol Biol Rep* **38,** 4715–4721 (2011).

7. Shahin, R. M. H., El Khateeb, E., Khalifa, R. H. & El Refai, R. M. Contribution of Toll-Like Receptor 9 Gene Single-Nucleotide Polymorphism to Systemic Lupus Erythematosus in Egyptian Patients. *Immunol. Invest.* **45,** 235–242 (2016).
